# Functional constraints in the evolution of brain circuits

**DOI:** 10.3389/fnins.2015.00303

**Published:** 2015-09-01

**Authors:** Conrado A. Bosman, Francisco Aboitiz

**Affiliations:** ^1^Cognitive and Systems Neuroscience Group, Swammerdam Institute for Life Sciences, Center for Neuroscience, Faculty of Science, University of AmsterdamAmsterdam, Netherlands; ^2^Facultad de Ciencias de la Salud, Universidad Autónoma de ChileSantiago, Chile; ^3^Departamento de Psiquiatría, Centro Interdisciplinario de Neurociencia, Escuela de Medicina, Pontificia Universidad Católica de ChileSantiago, Chile

**Keywords:** cortical evolution, canonical microcircuits, neuronal oscillations, predictive coding, cortical neurodevelopment

## Abstract

Regardless of major anatomical and neurodevelopmental differences, the vertebrate isocortex shows a remarkably well-conserved organization. In the isocortex, reciprocal connections between excitatory and inhibitory neurons are distributed across multiple layers, encompassing modular, dynamical and recurrent functional networks during information processing. These dynamical brain networks are often organized in neuronal assemblies interacting through rhythmic phase relationships. Accordingly, these oscillatory interactions are observed across multiple brain scale levels, and they are associated with several sensory, motor, and cognitive processes. Most notably, oscillatory interactions are also found in the complete spectrum of vertebrates. Yet, it is unknown why this functional organization is so well conserved in evolution. In this perspective, we propose some ideas about how functional requirements of the isocortex can account for the evolutionary stability observed in microcircuits across vertebrates. We argue that isocortex architectures represent canonical microcircuits resulting from: (i) the early selection of neuronal architectures based on the oscillatory excitatory-inhibitory balance, which lead to the implementation of compartmentalized oscillations and (ii) the subsequent emergence of inferential coding strategies (predictive coding), which are able to expand computational capacities. We also argue that these functional constraints may be the result of several advantages that oscillatory activity contributes to brain network processes, such as information transmission and code reliability. In this manner, similarities in mesoscale brain circuitry and input-output organization between different vertebrate groups may reflect evolutionary constraints imposed by these functional requirements, which may or may not be traceable to a common ancestor.

## Introduction

A noticeable feature observed in the central nervous system is its well-conserved organization across species. In vertebrates, pallial circuits (i.e., those in the superior aspect of the cerebral hemispheres) are functionally arranged through the interaction of excitatory and inhibitory neurons across multiple cortical layers (Lorente de No, 1938). According to this organization, excitatory neurons often have longer projections that allow the communication and information transfer between several brain areas and effectors. Inhibitory neurons have shorter projections, are mostly locally connected and are able to modulate excitatory forces, by imposing recurrent periods of neuronal inhibition, which are followed by transient windows of excitation (Isaacson and Scanziani, 2011; Kepecs and Fishell, 2014; Siegle et al., 2014). This reciprocal connectivity is at the basis of several computational mechanisms observed during brain functioning.

Remarkably, neurons do not connect randomly. Excitatory and inhibitory neurons are organized in relatively well-defined neuronal microcircuits, an organization that expands the computational possibilities of single units. Several comparative anatomical studies have consistently shown that these basic organizational principles are generally present across vertebrate classes and can be found across distant phyla, despite noticeable macroscopic anatomical differences (Shepherd, 2011; Ahumada-Galleguillos et al., 2015). This architectural stability has led some authors to consider this organization as canonical and to propose that these regularities are critical for sensory and cognitive processing (Douglas and Martin, 2004), a concept that traces back to the notion of “cortical unit” (cortical column, or mini-column) originally proposed by Mountcastle, and elaborated upon by Hubel and Wiesel. These early authors postulated the notion of a fundamental computational unit, upon which cortical functions could be elaborated incrementing the number of available units (Hubel and Wiesel, 1977; Gilbert, 1983; Mountcastle, 1997). However, an important—but yet unsolved—question to elucidate from an evolutionary perspective is whether a canonical microcircuit has evolved from a common ancestor or, alternatively, it represents a case of parallel or convergent evolution. In other words, what are the determinants of such canonical structure in evolution and are these determinants evolved from a common ancestor? In this article, we aim to outline an answer to these questions, presenting some ideas that may help to understand how it is possible to observe similar functional microcircuit architectures—despite substantial differences in macroscopic brain anatomy—, without the necessity to refer a common ancestor across different lineages.

Previously, we proposed that actual architectures of the mammalian brain rely on highly conserved neurodevelopmental mechanisms (Aboitiz and Montiel, 2007b; Bosman et al., 2014). Natural selection may have differentially modulated the expression and regulation of these neurodevelopmental mechanisms according to contingent adaptations, thus producing gross morphological differences across lineages (Aboitiz and Montiel, 2007b). Additionally, we suggested that a very basic excitatory-inhibitory interplay is a fundamental functional motif, which has been exploited through evolution to bear synchronized rhythmic activity through multiple brain architectures. Further, neuronal synchronization mechanisms might have evolved to support several neuronal computations, which are ultimately responsible of several high-level functions observed in the brain (Bosman et al., 2014; Womelsdorf et al., 2014). Here, we expand these previous concepts arguing that, despite neurodevelopmental differences produced by contingent adaptations, the canonical microcircuit organization is observed as a recurrent motif across evolution. This recurrence is the consequence of functional constraints imposed by the connectivity derived from canonical microcircuits. In turn, the compartmentalization of neuronal rhythms configures an optimized solution for advanced computational processing, a necessary adaptation for species to survive in an increasingly complex world.

Synchronization of cortical oscillations subserves several important cortical functions like gain control, postsynaptic coincidence detection of presynaptic spikes, phase coding, regulation of spike timing by inhibition, and routing of information among others (Fries, 2005, 2009; Singer, 2013; Bosman et al., 2014; Womelsdorf et al., 2014). Also, neuronal rhythm synchronization has been found consistently across different species and brain structures (Buzsáki et al., 2013; Bosman et al., 2014). Because of this ubiquity, some authors have considered synchronized oscillations merely a proxy for excitatory-inhibitory interactions (Merker, 2013; Ray and Maunsell, 2015), whereas others considered neuronal synchronization a fundamental computational principle (Fries, 2009; Bosman et al., 2014). Nevertheless, wide evidence sustains the notion that oscillatory phase-based relationships allow dynamic modulation in different brain structures (Engel et al., 2001; Salinas and Sejnowski, 2001; Varela et al., 2001; Fries, 2009; Bressler and Menon, 2010; Donner and Siegel, 2011; Singer, 2013; Bosman et al., 2014; Womelsdorf et al., 2014). Moreover, it has been recently proposed that neuronal oscillations can play a major role in predictive coding strategies (Bastos et al., 2012), which are pivotal in the implementation of inferential functionality in the brain (Rao and Ballard, 1999). From an evolutionary perspective, we argue that oscillatory synchronization may have been decisive in the evolution of cortical microcircuits. Oscillations may have imposed functional constraints to the circuitry architecture, and led to converge in canonical organization. Importantly, their acquisition may or may not be homologous across taxa. For example, in large-brained vertebrates, like mammals and birds, a shared canonical microcircuit may represent an ancestral condition, or alternatively, it may have emerged independently in both lineages. Whatever the case, we aim to show that the early acquisition of rhythmic synchronization patterns may have constrained the evolution of microcircuits and, in this manner been involved in the convergence of a particular canonical architecture. It is useful at this point to delineate the breadth of the concepts that we will discuss in this review. The term “mechanism” used in describing these circuits is primarily computational (e.g., communication through coherence), rather than synaptic (e.g., based upon plasticity of conductances). We used the term “constraint” as usually depicted in evolutionary contexts, normally interpreted as a stasis of features due to limited evolutionary plasticity, as opposed to a stasis of features due to common functional demands.

In the following sections, we will compare the multilayer organization in the brain between mammals and sauropsids (birds and reptiles together comprise a taxon called sauropsida). We will argue that those three lineages show a similar pattern of cortical connectivity, despite substantial differences in their neuronal development. Then, we will review how oscillations can emerge from a multi-layered organization and exert modulatory influences across cortical hierarchies, indicating a powerful functional constraint for this shared microcircuit.

## The canonical microcircuit in mammals and other species

**Figure 1:**
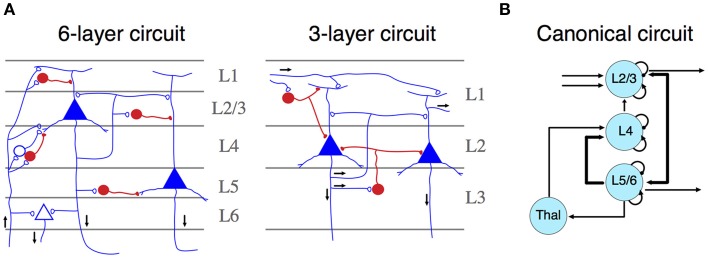
**(A)** Simplified representation of a six- (left) and three- (right) layer microcircuit. Neurons depicted in blue (open synapses) are excitatory cells, whereas neurons in red (close synapses) are inhibitory ones. Black arrows represent the flow of information across different layers. Both panels adapted from Shepherd (2011). **(B)** Schematic representation of a canonical microcircuit. Arrows represent connectivity within nodes, ordered spatially according to their anatomical localization. Curved arrows illustrate intrinsic (excitatory and inhibitory) connectivity. Adapted from Douglas and Martin (2004).

The mammalian isocortex is part of the pallium—the “roof” of cerebral hemispheres—that also includes the hippocampus, the olfactory cortex and parts of the amygdala. Lorente de No early established that despite number, cell form and size variations, the structural details of the isocortex remains constant across species (Lorente de No, 1938). Contrasting with other pallial regions, the isocortex is characterized by a six-layered organization, characterized by a central layer (layer 4, L4), containing inhibitory and excitatory neurons that receive most of the thalamic input (Figure 1A). These neurons target mostly interneurons and fibers of layers 1 and 2, providing feedforward inhibition to the cortico-cortical connections present in this layer (Shepherd, 2011). L2 and L3 contain pyramidal cells that receive synaptic inputs from local interneurons and excitatory neurons from L4. Their axons project to other cortical regions. L1, L2, and L3 comprise the supragranular layers. Conversely, L5 and L6 encompass infragranular layers. L5 contains large pyramidal cells, which project to subcortical structures or, as in the motor cortex, to the spinal cord trough the internal capsula. L6 provides efferent connections to the thalamus via small pyramidal cells. Both layers receive synaptic inputs from collateral projections of L3 neurons and inhibition by local interneurons (Lorente de No, 1938; Shepherd, 2011; Harris and Shepherd, 2015).

Neurons configuring this circuit are organized into radial columns of clonally related cells that cross all these layers (Mountcastle, 1997; Noctor et al., 2001). Furthermore, sibling neurons within a cortical column are preferentially interconnected among themselves, showing similar stimulus feature selectivity (Li et al., 2012); and this microcircuit assembly is mediated by transient electrical couplings among sister neurons (Yu et al., 2012). More recently, it has been found that the unique inside-out developmental gradient of the mammalian isocortex, partly determined by the reelin signaling pathway, is a key regulator of this lineage-dependent columnar microcircuit (He et al., 2015). This evidence indicates that the assembly of the isocortical canonical microcircuit is strongly dependent on developmental factors unique to mammals, even if there are general patterning mechanisms that are shared with other vertebrates (Aboitiz, 2011). Thus, the specific development of similar circuitries in other amniotes may rely on different, but convergent developmental mechanisms. Functionally, cortical columns depict clear excitatory-inhibitory relationships across neuronal constituents, which facilitates information transfer processes and oscillatory dynamics. In the original description of canonical microcircuits (Figure 1B), Douglas and Martin (1991) aimed to explain how transient stimulation of the visual cortex of the cat produced cortical fast excitatory currents followed by slow, long-lasting inhibition. They described a model using intrinsic excitatory-inhibitory relays observed in L4 are able to modulate transient activities derived from thalamic inputs thus providing major substantial excitation, which can be transferred to infra and supragranular layers, where further processing beyond pulsatile stimulation activity can take place (Douglas and Martin, 1991). Furthermore, dynamic canonical microcircuits based in the same inhibitory-excitatory relationships have been related to several important computational processes (Bosman et al., 2014; Womelsdorf et al., 2014). For instance, canonical microcircuits architectures are relevant implementing feedback and feedforward inhibition. Feedback inhibition has been implicated in the origins of high-frequency oscillations (Cardin et al., 2009; Siegle et al., 2014). Conversely, feedforward inhibition has a major role implementing gain control and divisive normalization (Wilson et al., 2012). These computational processes are at the basis of several important sensorial and cognitive functions (Bosman et al., 2014; Womelsdorf et al., 2014), and canonical microcircuits provide a basic connectivity motif that accounts for these computations (Douglas and Martin, 2004; Shepherd, 2011).

Yet, this description leaves unanswered the question whether this architecture derives from a primitive ancestor common to other vertebrates. This question can be addressed by comparing the mammalian microcircuit architecture with those observed in birds, reptiles (sauropsids, the sister taxon of mammals) or perhaps more important, modern amphibians, whose brains are morphologically more similar to that of the putative common amniote ancestor (shared common ancestor with sauropsids and mammals). Unfortunately, there is yet little evidence on circuit organization in amphibians, and we will have to rely on evidence recently gathered in reptiles and birds.

### Connectivity and development of mammalian and sauropsidian brains

The isocortex has six layers and radial input organization. It differs in its overall organization from other cortices like the hippocampal region and the olfactory cortex, which display a three-layered organization and a tangential organization of inputs, (Figure 1A) (Nieuwenhuys, 1994). In reptiles, some cortical structures fit the design of three-layered structures. However, other pallial components, namely the dorsal ventricular ridge (DVR), depict a nuclear appearance, resembling parts of the mammalian amygdalar complex. In birds, the DVR is roughly subdivided into a nidopallium and a mesopallium among other structures. Comparing with other regions of the pallium, it becomes a highly differentiated structure that comprises much of the auditory and visual sensory inputs to the brain. This projections make the DVR complex the main sensory processing structure in birds (for review see Aboitiz and Montiel, 2007b).

Despite the observed differences in laminar vs. nuclear organization of the mammalian isocortex and the avian nidopallium, respectively, Harvey Karten described in 1960's similar processing circuits in these two structures. These similarities led Karten to postulate the “equivalent cell hypothesis,” asserting that this circuit was homologous in birds and mammals (Karten, 1968, 1969). From this hypothesis, it follows that the nidopallium of birds is homologous to parts of the mammalian isocortex, i.e., both derive from a same structure in a common ancestor (Karten, 2013, 1997), an assertion that has been recently challenged (see below). Recent studies have provided some evidence that can be interpreted in favor of this hypothesis. For instance, auditory circuits in birds depict sensory thalamic projections targeting a region termed Field L2, which correspond to the isocortical L4. L2 neurons project to Field L1 (and the caudal mesopallium), and to L3, corresponding to supragranular and infragranular isocortical layers, respectively (Wang et al., 2010), mimicking the canonical organization observed in columnar circuits. A similar “columnar-type” organization has been described in the visual DVR of the chick (Ahumada-Galleguillos et al., 2015). Recently, Calabrese and Woolley (2015) investigated the electrophysiological properties of the auditory DVR in birds, and compared this evidence with the known properties of different isocortical layers. They observed similarities between birds and mammals in the latencies, noise correlations, and coding strategies of the different components of this microcircuit. Additionally, it was observed that afferent connections of neurons projecting to the thalamus express similar neurochemical markers (i.e., EAG and RORB) in different pallial regions of mammalian, reptilian, and avian brains. Conversely, output projection neurons of different pallial regions express the marker Er81 in both mammals and sauropsids (Dugas-Ford et al., 2012). However, it should be noted that while this evidence compellingly indicates the existence of common input-output neuronal phenotypes in different pallial regions across amniotes, it does not necessarily imply that the avian nidopallium is homologous to the mammalian isocortex as a region (Aboitiz and Zamorano, 2013).

The above interpretation has been challenged by some authors who argue that the avian nidopallium (and mesopallium) and the mammalian isocortex have different developmental origins, i.e., the isocortex derives from embryonic dorsal pallial components while the nidopallium and mesopallium derive from ventral and lateral pallial components (Aboitiz, 1992, 1995; Striedter, 1997; Fernandez et al., 1998; Puelles et al., 1999, 2000; Medina and Abellán, 2009). More recently, it has been suggested that the isocortex shares with the avian nidopallium a common genetic determinant—tentatively driven by a Pax6-dependent cascade (Georgala et al., 2011), and the expansion of both structures is largely based on the amplification of similar genetic mechanisms (Aboitiz and Montiel, 2007a; Aboitiz, 2011; Aboitiz and Zamorano, 2013). This evidence suggests the existence of a continuous overlap of dorsal and ventral morphogenetic signals that drives the regional differentiation of pallial regions (Hoch et al., 2009), rather than parcellating the embryonic pallium in discrete components. Furthermore, these morphogenetic signals have been differentially modulated in mammals and sauropsids, resulting in the expansion of the DVR in the ventral and lateral pallium of sauropsids, and in the expansion of the isocortex in the dorsal pallium of mammals, respectively (Aboitiz, 2011; Aboitiz and Zamorano, 2013). Similarly, Luzzati and coworkers (Luzzati et al., 2009; Luzzati, 2015) have advanced the hypothesis that the emergent isocortex of early mammals co-opted genetic pathways involved in lateral pallial (i.e., olfactory cortex) differentiation and activated them in the neocortical proliferative epithelium to yield the supragranular neuronal phenotypes.

Thus, the weight of the developmental and genetic evidence indicates that the mammalian isocortex and the avian nidopallium originated as expansions of different embryonic regions present in the common ancestor, possibly through differential amplification of telencephalic signaling centers that are shared in both taxa. This, again, is consistent with structural and functional convergence between these structures, rather than homology.

### Tangential networks in the isocortex and other cortices

Another approach regarding the ancestral circuitry of mammals and sauropsids (in this case, reptiles) has highlighted the similarities in tangential organization of the mammalian isocortex and olfactory cortex, together with that of the reptilian cortical structures (Lynch, 1986; Shepherd, 2011; Rowe and Shepherd, 2015). In this scenario, the isocortex is primarily a tangentially associative network, where afferents were ancestrally located in the superficial marginal zone, running parallel to the cortical surface and contacting several pyramidal cell apical dendrites in tandem. The now characteristic radial, columnar isocortical organization was possibly a late innovation, concomitant with the differentiation of primary sensory areas and the development of the subplate. The subplate served as a substrate for thalamic axonal growth in the white matter underlying the cortical plate (Aboitiz et al., 2005). In line with this hypothesis, several authors have very recently highlighted striking connectional and functional similarities between the mammalian olfactory cortex and the reptilian dorsal cortex (the latter deriving from the dorsal pallium, and the likely regional homolog of the mammalian isocortex), both exhibiting similar laminar organization and an apparent poor topographic mapping of the sensory surfaces (Fournier et al., 2015; Naumann et al., 2015; Rowe and Shepherd, 2015). This indicates a shared combinatorial and associative array in both structures. Early studies in the isolated dorsal cortex of the turtle describe intrinsic circuit properties that resemble very much those observed in mammalian isocortex. Compared with mammalian isocortex, the dorsal cortex circuitry is simpler. It consists basically in two types of neurons, pyramidal and stellate cells. Thalamic inputs usually target pyramidal cells to elicit volleys of excitatory activity that it is further controlled by feedforward inhibition (Smith et al., 1980). Remarkably, intrinsic long-lasting inhibition is observed after stimulation (Kriegstein and Connors, 1986), similar to those responses observed in cat visual cortex and other mammals (Douglas and Martin, 1991; Shepherd, 2011). Furthermore, Fournier et al. (2015) called attention to the oscillatory activity of cortical networks in both reptiles and mammals, emphasizing activity in the beta range (15–35 Hz), which in the olfactory cortex appears to be involved in discrimination learning and pattern completion, while in the reptilian dorsal cortex has been tentatively associated to spatial processing. These authors suggest that beta frequencies are involved in long-range networks that participate in coding for stimulus selectivity. This evidence suggests a likelihood of convergence over strict homology and it is in agreement with our original proposal about the consequences of the development of an associative olfactory-hippocampal in the origin of the laminar isocortex (Aboitiz et al., 2003; Aboitiz and Zamorano, 2013). Particularly, we consider the use of the olfactory-hippocampal axis as an interface of the dorsal pallium in reptiles. When as it expanded, it was able to recruit different sensory systems in this network.

### How complex were the ancestral microcircuits?

The analysis of neurodevelopmental constraints unveils two possible mechanisms that can explain the evolution of the isocortex. So far, the evidence for a common microarchitecture in the avian and mammalian brains suggests the possibility of an ancestral microcircuit present in pallial structures, of all amniotes. However, the alternative explanation of evolutionary convergence is also likely. In both scenarios, the architectural circuit stability of a canonical microcircuit may be the result of phylogenetically parallel elaborations on a quite simple, basic input-output organization driven by functional and/or developmental constraints, as there are not many ways to perform early processing of sensory input, and there are not many developmental or genetic pathways to achieve this organization. It is therefore important to elucidate the specific characteristics of this putative ancestral circuit.

Some macroscopic features of a primitive telencephalon may help to understand the organization of a very simple ancestral circuit. The rudimentary telencephalon of early amniotes was a quite a small tubular structure (Kielan-Jaworowska et al., 2004; Rowe and Shepherd, 2015), perhaps more similar in morphology to the telencephalon of present amphibians, who display a very limited degree of radial neuronal migration and a conspicuous tangential arrangement of inputs in the superficial or molecular layer. Furthermore, both within therians (placental and marsupial mammals) and within sauropsids, an increase in complexity can be observed from more basal forms to more derived forms, associated with the development of an embryonic subventricular zone housing intermediate progenitor neurons (Cheung et al., 2010). Therefore, it is quite likely that this basic processing circuit became increasingly complex independently in both lineages, concomitant with larger brain sizes and more complex behaviors.

A basic characteristic of the canonical microcircuit is the balanced interplay between excitation and inhibition (van Vreeswijk and Sompolinsky, 1996; Isaacson and Scanziani, 2011). This balanced activity represents the basis of complex neuronal responses embedded in microcircuits (Salinas and Sejnowski, 2001; Tiesinga and Sejnowski, 2009; Isaacson and Scanziani, 2011; Womelsdorf et al., 2014). Inhibitory interneurons may have served to regulate the oscillatory dynamics of such primordial circuits (Tiesinga and Sejnowski, 2009). Accordingly, a rudimentary circuit architecture, organized through input receiving and output sending neurons, with intermediate associative excitatory neurons and inhibitory interneurons providing feedforward and lateral interactions, is very likely to have existed in pallial regions of the ancestral amniote (see also Rowe and Shepherd, 2015). It is possible though, like in the reptilian cortex, that input and output neurons were tangentially separated (Dugas-Ford et al., 2012). Although maintaining this same general architecture, the processing microcircuits of the mammalian isocortex, the nido- and the mesopallium of birds are very likely much more complex than this, including larger numbers of excitatory and inhibitory interneurons, compartmentalization of information and well-organized interareal communication.

### Large scale organization of mammalian microcircuits

In larger brain sizes, as it is observed in mammals, canonical microcircuits are embedded in hierarchically organized neuronal networks. Remarkably, adjacent areas shown strong regularities in their laminar organization and interareal connectivity, a feature recently observed in recent anatomical studies of primate visual cortex, using retrograde tracers combined with electrophysiological techniques (Markov et al., 2014). These studies depict a basic organization of the connectivity of microcircuits across areas. Feedforward projections originate in neurons of supragranular layers of “lower-order” areas (i.e., V1 or closer to it) and target granular neurons of L4 of “higher-order” areas (i.e., successively farther from V1) (Lund, 1988; Felleman and van Essen, 1991; Markov et al., 2014). Conversely, feedback projections depart from infragranular layers of higher order areas, to end in the proximities of L4 of lower order areas (Markov et al., 2014). Based on these regularities, several attempts of modeling these anatomical networks have been performed (Felleman and van Essen, 1991; Markov et al., 2014). Recent models have emphasized the existence of a *bow-tie* network architecture with a processing core—areas that share connections for multiple origins—with several independently connected sensory areas (Ercsey-Ravasz et al., 2013). In this model, interareal connectivity patterns are compatible with both, long-range connection distribution and local microcircuit architectures (Markov et al., 2013). Alternative models of organization emphasize a *small-world* network architecture, in which hierarchies are distributed across hubs or regions receiving a high number of connections (see Bullmore and Sporns, 2009). These features facilitate wire-length minimization in concomitance with increasing communication efficiency, leading to an overall increase in neocortical computations associated with a reduction in energy consumption (Bullmore and Sporns, 2012; Ercsey-Ravasz et al., 2013; Markov et al., 2014). This leads us to the hypothesis that these computational advantages may have been functionally constrained the evolution and convergence of these cortical hierarchies across phyla.

## Dynamic activity of laminar microcircuits

**Figure 2:**
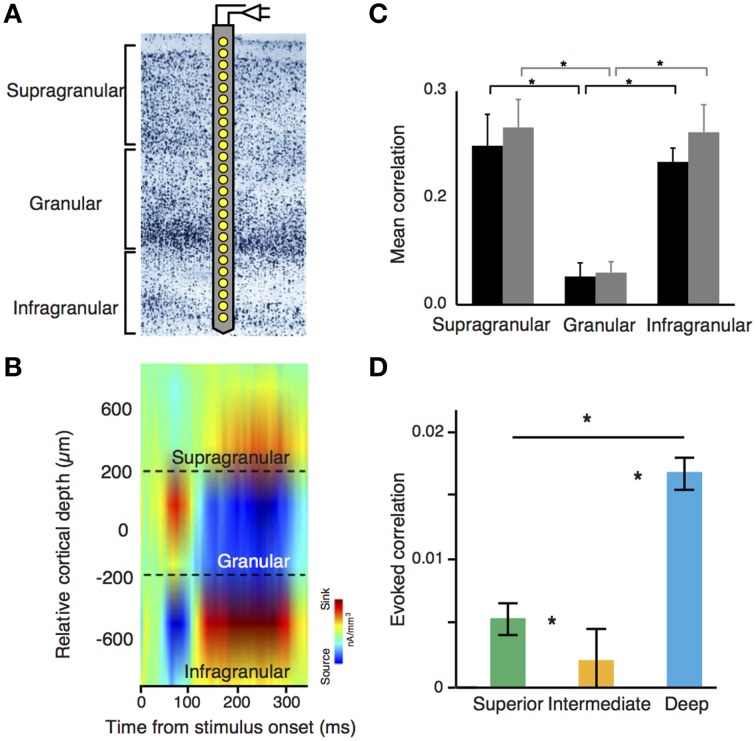
**(A)** Laminar recording on visual cortex using a linear microelectrode array (LMA). **(B)** Stimulus-evoked current source density (CSD) analysis using laminar probes. LMAs typically depict sinks (cation inflow from the extracellular to the intracellular space, in red) and sources (cation outflow from the intracellular to the extracellular space, in blue) of neuronal activity. This configuration pattern is useful to segregate differential layer activity. **(C)** Noise correlation amplitude is significantly lower in granular layers as compared with supra and infragranular layers. Black and gray bars denote two different monkeys. **(B,C)** adapted from Hansen et al. (2012). **(D)** Decrease of noise correlations amplitude in cyto-architectonically defined intermediate layers of the songbird auditory caudal meso/nidopallium cortex [Asteriks indicate significant differences in correlations between regions (*p* < 0.05, Kruskal-Wallis test with multiple comparisons correction)]. Adapted from Calabrese and Woolley (2015).

The understanding of the neuronal dynamics generated in canonical microcircuits has been facilitated by the popularization of techniques that enable simultaneous recordings through multiple areas and cortical layers (Lewis et al., 2015). Linear microelectrode (LMAs) feature several contact points through one or multiple shanks (Figure 2A). This configuration facilitates recordings of neuronal activity—spikes and local field potentials (LFP)—simultaneously across layers. In animals, high-density electrocorticograms (ECoGs) arrays can be used to study cortical LFP-LFP interactions across different brain areas. In LMAs, LFPs are usually studied using current source density (CSD) analysis, a technique amendable to give access to the sinks and sources of voltage differences at the extracellular space (Mitzdorf, 1985). CSD analysis can be used to identify electrode position based on the different profiles obtained at different layers (Figure 2B). Additionally, the temporal coordination between spikes and LFPs can be described using spike-field coherence based techniques, which quantify the phase relationships between the ongoing LFP and spike activity. All these techniques are especially advantageous for the study of long-range interactions across cortical microcircuits (Lewis et al., 2015).

### Noise correlations and dynamic structure of microcircuits

Many of the studies using LMAs have focused on understanding the dynamics of neuronal assemblies using noise and stimulus correlations, which are two important measures of the conjoined variance among neurons (Averbeck and Lee, 2004). Noise correlations quantify the common variance of a neuronal population that cannot be explained by any external input, representing a default “common response” from a particular neuronal population. Since noise correlations are very much dependent of the anatomical connectivity pattern through the laminar cortex, these measurements can be used to disentangle the basic functional connectivity across neurons. In mammals, it has been well observed that the intensity of noise correlations is not equally distributed across all layers and cell types (Ecker et al., 2010; Renart et al., 2010; Hansen et al., 2012; Smith et al., 2013). In rodents and primates, L2/3, L5, and L6 show high intensity of noise correlations. In contrast, L4—the main target of thalamic projections—shows little or insignificant amounts of noise correlations, and correlations between interneurons are stronger than those between pyramidal cells in both supra and infragranular neuronal populations (Hansen et al., 2012; Smith et al., 2013) (Figure 2C). Notably, this organization pattern seems to be present in circuits from auditory DVR nuclei in birds. Here, noise correlations are stronger in “superficial” and “deep” layers of the nuclei, but weak in the intermediate ones (Calabrese and Woolley, 2015) (Figure 2D). This profile is also consistent with previous proposals about the origin of noise correlations. In mammals, cortical horizontal connections—more abundant in supra and infragranular layers—are responsible of noise correlations (Ecker et al., 2010; Renart et al., 2010). In both, avian and mammalian laminar structures, deep layers or their equivalents are densely interconnected and both groups show stronger spontaneous correlations between interneurons than those observed in pyramidal cells (Calabrese and Woolley, 2015).

Importantly, the functional connectivity denoted by noise correlations can change according to the brain state of the animal. During neuronal development, noise correlations decrease its intensity as a function of aging. This decrease is accompanied by an increase of the sparseness of neuronal responses, which are dependent upon the experience acquired by the animal (Smith et al., 2015). Spontaneous activity can be less correlated once the subject is engaged in a sensory-driven task, as it is in the case in visual attention or during arousal (Vinck et al., 2015). Furthermore, arousal status of the animal can decrease the intensity of spontaneous correlations and firing rates (Vinck et al., 2015), leading to greater sparseness of neuronal responses and being indicative a change of the underlying network state. These findings indicate how noise correlations are affected if different areas can coordinate and communicate during different brain states.

In sum, noise correlations are useful to understand neuronal dynamics taking into account anatomical connectivity across different cortical layers. Mammals and birds show striking resemblances in the distribution of noise correlations across layers and neuronal types involved. These findings are compatible with canonical microcircuit architectures in these different taxa, and suggest common functional requirements. Nevertheless, a full characterization of the functional dynamics during different brain states of the animal should take in consideration the intra- and inter-laminar oscillatory dynamics.

### Neuronal oscillations and microcircuits

Neuronal oscillations can be consistently related across several brain structures and species in a similar fashion (Buzsáki et al., 2013; Bosman et al., 2014). These relationships can be traced back to a limited set of circuit motifs, which are in turn, strongly dependent on the inhibitory-excitatory interplay presented in cortical and subcortical microcircuits (Bosman et al., 2014; Womelsdorf et al., 2014). Therefore, it is possible that neuronal oscillations may have conferred advantages for low-level system processing functions throughout evolution, and may also explain why neuronal oscillations are conspicuously found in several brain structures (Bosman et al., 2014).

Laminar recordings have consistently shown that rhythms at different frequencies are highly compartmentalized across layers. In hippocampus—a three-layer structure, resembling ancient brains—gamma oscillations (30–90 Hz) are functionally separated in two different bands (slow- and medium/fast gamma-band oscillations) that may have different properties and relate differently to the more prominent hippocampal theta waves. Slow gamma synchronizes between hippocampal areas CA3 and CA1, whereas medium/fast gamma is synchronized to rhythmic activity in the medial entorhinal cortex (Bragin et al., 1995; Colgin et al., 2009). In the three-layer DVR of turtles, a preeminence of highly coherent beta oscillations has been notified (Prechtl et al., 1997, 2000). This oscillatory activity resembles similar dynamics observed in the mammalian three-layer piriform cortex (Fournier et al., 2015). In birds, fast oscillatory bursts (500–600 Hz) are mostly generated by cells located in the outer layers of the optic tectum (OT), which mirrors that observed in the mammalian lateral geniculate nucleus (Marín et al., 2005). LFP recordings of the OT have shown a different oscillatory profile across layers. Whereas superficial layers of the OT show low gamma band oscillations, deep OT layers display three recognizable bands (alpha, low gamma, and high oscillations) (Sridharan et al., 2011). Importantly, these gamma oscillations are mostly locally generated and the microcircuit architecture and neuronal involvement underlying this generation are similar to those described in mammals (Goddard et al., 2012). Since OT neurons project to different pallial structures, these differences may have deep consequences in the implementation of avian cognitive abilities such as attention and visual discrimination (Sridharan and Knudsen, 2015). To the best of our knowledge, no specific studies linking LFP oscillation dynamics and pallial structures have been performed yet. Nevertheless, the similarities in anatomical connectivity and noise correlations pattern described by Calabrese and Woolley (2015) are highly indicative of similar oscillatory profiles. In mammalian visual areas, gamma oscillations have been consistently found in supragranular layers (Buffalo et al., 2011; Xing et al., 2012; Roberts et al., 2013; van Kerkoerle et al., 2014), while alpha (8–12 Hz) and beta have been recorded in infragranular layers (Lopes Da Silva and Storm Van Leeuwen, 1977; Bollimunta et al., 2008; Buffalo et al., 2011; Spaak et al., 2012; van Kerkoerle et al., 2014), a finding compatible with earlier *in vitro* studies showing pyramidal cells spontaneously oscillating at 12 Hz in this layer (Silva et al., 1991).

**Figure 3:**
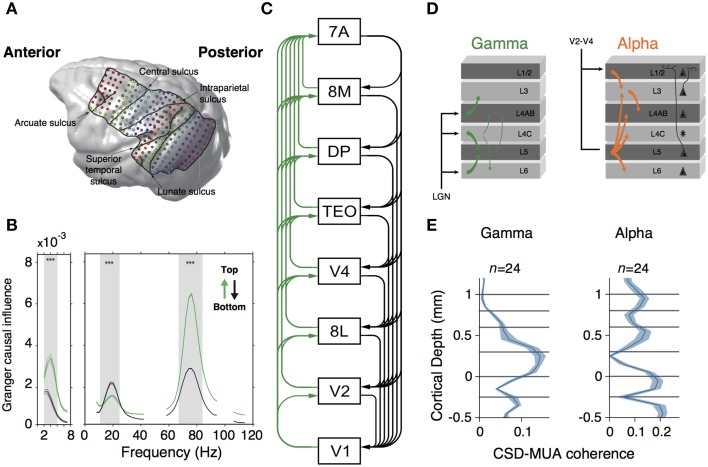
**(A)** MRI-scan based rendering of a monkey brain implanted with an ECoG grid. Lines indicate the boundaries of recorded regions. Dots indicated 252 recording sites. **(B)** Granger-causal influence spectra of across all regions of interests separated for top-down (black line) and bottom-up (green line) directions. Spectral top-down influences are mostly at beta frequency-band, whereas bottom-up influences occurs at gamma frequency-band. Asterisks indicate significant differences (*p* < 0.001, randomization test). **(C)** Hierarchical ranking of recorded areas according to Markov et al. (2014). Green lines indicate bottom-up influences. Black lines denote top-down influences. **(A–C)** are modified from Bastos et al. (2015b). **(D)** Representation of Granger causality influence across layers for gamma (left) and alpha (right) frequency bands. Gamma causal influences depart from L4 to infra and supragranular layers. Alpha is mostly observed in infragranular layers and influences granular and supragranular layers. **(E)** Laminar profile of spike-field coherence (measured through the CSD-MUA coherence) for gamma (left) and alpha (right) frequency band. Middle and supragranular layers show increased gamma field coherence whereas alpha is concentrated in infragranular layers. **(D,E)** are modified from van Kerkoerle et al. (2014).

What could be the advantage of having compartmentalized oscillations? In the isocortex, synchronization of local neuronal assemblies can lead to rhythmic synchronization across cortical regions (Buschman and Miller, 2007; Gregoriou et al., 2009; Bosman et al., 2012; Salazar et al., 2012; Jia et al., 2013). If interareal synchronization can serve as a mechanism of dynamic communication across brain areas (Fries, 2005; Bosman et al., 2012), then compartmentalized oscillations may contribute to segregate the information received from feedback and feedforward projections on a given area. This hypothesis has been evaluated in two recent studies (Figure 3). In the first study, Bastos and colleagues measured spectral Granger causal influences across eight areas of the visual hierarchy, using intracranial electrocorticographic recordings in non-human primates engaged in a visual task (Bastos et al., 2015b) (Figure 3A). They observed asymmetrical influences of the directionality of different frequency bands. Gamma-band influences were mostly feedforward, whereas beta oscillations exerted feedback influences across brain areas (Figure 3B). Strikingly, these spectral asymmetries configured a dynamical hierarchy that correlates with the anatomical hierarchy of the explored areas (Markov et al., 2014) (Figure 3C). In the second study, van Kerkoerle et al. (2014) implanted LMA in areas V1 and V4 of monkeys trained in a figure-ground discrimination task. They used Granger causality, microstimulation techniques and pharmacological blockade of NMDA receptors to convincingly demonstrate that gamma-band activity started its influence in the granular layers within a column, after which it propagated to the superficial and deeper layers (Figure 3D). Conversely, alpha-band activity triggered in superficial and deeper layers targeted granular layers (van Kerkoerle et al., 2014) (Figure 3E).

Despite the fact that the two frequency bands observed in the infragranular layer in the Bastos et al. (2015b) and the van Kerkoerle et al. (2014) studies are different, both suggest a putative role of oscillatory compartmentalization through cortical layers. Low frequency oscillations may convey top-down signals and exert modulatory influences downstream the cortical hierarchy. Conversely, local gamma-band oscillators may convey bottom-up modulatory signals to influence cortical activity upstream cortical hierarchy. Also, it is yet unknown how information conveyed by gamma and low-frequency oscillations can be integrated in cortical microcircuits. Perhaps, cross-frequency coupling mechanisms (von Stein et al., 2000; Canolty and Knight, 2010) may play a major role during integration. Regardless the specific underlying mechanisms involved in these processes, this specific connectivity may facilitate the implementation of specific coding strategies across cortical regions, as we will discuss in the following section.

### Coding strategies within microcircuits

What are the basic computations supported by a canonical microcircuit? Canonical microcircuit architectures support the implementation of predictive coding and causal modeling processing (Friston, 2010; Bastos et al., 2012; Adams et al., 2013). Predictive coding based architectures can optimize information transfer to different areas based on generative models of a priori predictions and error estimation. Error estimations originated in lower areas can accumulatively correct and generate subsequent predictions. These predictions, in turn, modulate signal acquisition in early sensory cortices (Friston, 2010). As it is described by Friston and colleagues (Friston, 2010; Adams et al., 2013), error predictions are implemented in systems entailed to reduce the “free energy,” an information theory concept related to the level of self-information (surprise) associated with an event (e.g., sensory data). Self-sustained biological systems (as the brain) tend to reduce the surprise associated to environmental changes, preserving their physiological variables constant across multiple changes. Thus, free energy minimization is the actual consequence of prediction error minimization. Interestingly, such models require a laminar compartmentalization to work optimally. Because free energy minimization is a homeostatic response, the conserved canonical microcircuit would set the basis for acute adaptation to uncertainty in a volatile environment.

Furthermore, a free energy minimization model can be implemented through the operation of neuronal oscillations through different cortical layers (Bastos et al., 2012). The studies of Bastos et al. (2015b) and van Kerkoerle et al. (2014) seem to suggest that compartmentalized oscillations may play a role in the implementation of predictive coding strategies in a canonical microcircuit. Their findings also suggest that low frequency oscillations such as beta- or alpha-band may convey prior prediction signals and exert their modulation in a top-down fashion. Inversely, high frequency oscillations such as gamma-band may bottom-up communicate error signals to higher cortices.

These hypotheses were tested in two recent studies that used dynamic causal modeling (DCM), a neural mass model that use predictive coding functions to mimic canonical microcircuits (Pinotsis et al., 2014; Bastos et al., 2015a). Pinotsis et al. (2014) were able to reproduce stimulus contrast dependences in neuronal responses and track their origins to the pyramidal neurons with forward projections. In the study of Bastos et al. (2015a), a DCM model implementing feedback-feedforward beta-gamma asymmetries between V1 and V4 replicated previous experimental observations obtained in monkeys during a selective attention task (Bosman et al., 2012). In both studies, the manipulation of the strength of synaptic connectivity and the excitatory-inhibitory balance across cortical columns provided critical evidence about the role of compartmentalized oscillations in the generation of both predictive coding strategies and transfer spectral functions through cortical microcircuits.

Altogether, these pieces of evidence raise an interesting question. Are the observed microcircuit similarities between different phyla a reflection of functional constraints imposed by the same predictive coding strategy? So far, no studies have tested this hypothesis, since direct comparisons between species are always difficult to establish. Nevertheless, comparative anatomical studies in homologous areas would help to identify which neuronal types and what type of connections organizes microcircuit architectures, and functional studies emphasizing the use of analogous and comparable sensorial and cognitive tasks might unveil many of the functional similarities observed between different species. Importantly, predictive coding strategies are ubiquitous in several brain areas and, as neuronal rhythms, are linked to many cognitive functions (Friston, 2010). Recently, it has been observed a link between the conserved canonical microcircuit observed in sensory areas with the asymmetry observed in the motor cortex of primates (Adams et al., 2013). This asymmetry and the computational implications for active inference (namely proprioceptive predictions) are described by Adams et al. (2013). It remains to be tested whether the stability of canonical microcircuits across evolution is related to the implementation of predictive coding strategies based on the compartmentalization of neuronal oscillations.

## Discussion

In summary, neurodevelopmental and anatomical studies suggest a parallel evolution for canonical microcircuits. This evolution may be traced back to a common ancestor, although there is no compelling evidence supporting this claim. Despite the evolutionary distance between different taxa, the microcircuit architecture seems to be well conserved across species. Here, we aimed to explain this similarity, proposing that functional properties of the microcircuit have conferred evolutionary advantages that predisposed the selection of this particular architecture, even in the presence of different evolutionary contexts. We also claimed that the elementary functions derived from these canonical microcircuit architectures, namely the presence of compartmentalization of functions and neuronal oscillations, are derived from the basic excitatory-inhibitory interplay, which is a functional hallmarks of this evolutionary stability. Finally, we postulated that a basic neuronal architecture motif—proposed as a minimalist canonical microcircuit—might represent an early evolutionary solution to optimize the use of the predictive coding strategies that extent isocortical computational capacities. In other words, we expand the concept of a canonical microcircuit from just reflecting an ancestral condition, to become a pivotal functional motif in brain evolution across species. Thus, the functions of the canonical microcircuit across species support the notion of strong functional constraints associated to oscillatory activity in the evolution of the laminar or laminar-like pallium. Nonetheless, it must be noted that the developmental processes involved in the generation of canonical microcircuits may be quite different across amniotes (He et al., 2015). This suggests that this ancestral pallial circuit has been subjected to different embryological transformations in sauropsids and mammals, in order to maintain its basic architecture in the context of increasing brain size and circuit complexity but strikingly has converged into a more or less similar architecture able to support fundamental computational processes.

The ancestral canonical microcircuit can be reconstructed focusing on simpler circuit architecture of basal tetrapods like amphibians, which may better resemble the ancestral amniote condition. The functionality and connection pattern of canonical circuits in rudimentary tetrapods can contribute to unveil the principles that explain brain complexity and to understand the evolution of highly derived brains like those of mammals and birds. Moreover, a role of high frequency oscillation in sensory processes—such as odor identification and rudimentary visual processing—has been previously described in arthropods and cephalopods, among other species (cfr., Table 1 of Bosman et al., 2014, see also Bullock and Basar, 1988; Kirschfeld, 1992; Stopfer et al., 1997), providing further support for convergent origins of dynamically-balanced microcircuits.

Finally, in the context of the discussion regarding the homology or convergence of amniote canonical microcircuits, it is interesting to refer the convergent columnar microarchitecture in the retinae of mammals and flies. This did not pass unnoticed to Ramón y Cajal, who imagined a common circuit that maintained the main features of both visual systems (Cajal and Sanchez, 1915; Sanes and Zipursky, 2010). In both groups, there is a vertical arrangement consisting of three processing layers (with two sequential synapses) before the inputs leave the retina: (i) photoreceptors synapse on (ii) bipolar cells (lamina neurons in flies) that in turn feed onto (iii) ganglion cells (transmedullary neurons in flies). In both synaptic relays, mammals and flies show strong horizontal connections that modulate the vertical transmission of inputs. Axons from ganglion cells or their fly equivalents leave the retina and project to a relay center (thalamus/midbrain and lobula complex, respectively) before reaching the telencephalon/thalamus in mammals, or the protocerebrum in flies. Although these similarities suggest that common ancestor of flies and mammals would have a complex retina, there are several reasons that preclude this option: First, the common ancestor of chordates had a brain more likely similar to that of cephalochordates, consisting of a spot of pigment cells connected directly with cerebral centers through a projection neuron, with no relay stations or signs of horizontal interactions. Second, other basal deuterostomes (hemichordates) show definitely no evidence of anything resembling a retina (Lacalli, 2004; Aboitiz and Montiel, 2007b; Suzuki et al., 2015). Third, an increase in synaptic retinal complexity can be observed within vertebrates, where the more basal agnathans (petromyzonts) display a rudimentary, two-layered retina (with receptors synapting directly ganglion cells, similarly to cephalochordates). In more advanced adult agnathans (cyclostomes) and in jawed vertebrates the retina becomes three-layered by the introduction of a bipolar cell layer between receptors and ganglion cells. Remarkably, the ontogeny of this circuit follows the same sequence as in phylogeny (Lamb, 2013). This is a good example of tight similarity due to convergence based on functional demands, in which a (retinal) canonical microcircuit has evolved independently in two different lineages, nonetheless being based on homologous, Pax-6 dependent patterning mechanisms (Gehring and Ikeo, 1999; note that this gene is also important for telencephalic patterning; see above). However, like in the mammalian isocortex, the specific mechanisms involved in the generation of similar circuits might be different in insects and vertebrates. Furthermore, ontogeny seems to follow similar steps as those acquired in phylogenetic history. Similarly, conserved pallial canonical microcircuits might be consequence of common processing requirements more than representing an ancestral condition.

Many homologous characters (like fins and hands) retain their identity despite serving different functions; however, in the case of canonical microcircuits we observe that function and structure conflate in a common phenotype, making it difficult to dissociate homology from functional convergence. In these conditions, we claim that a better approach to unveil the ancestral condition is one that combines comparative structure, function, development, and genetics.

### Conflict of interest statement

The authors declare that the research was conducted in the absence of any commercial or financial relationships that could be construed as a potential conflict of interest.
